# Associations of serum calcium levels and dietary calcium intake with incident type 2 diabetes over 10 years: the Korean Genome and Epidemiology Study (KoGES)

**DOI:** 10.1186/s13098-018-0349-y

**Published:** 2018-06-19

**Authors:** Kyoung-Nam Kim, Se-Young Oh, Yun-Chul Hong

**Affiliations:** 1 Division of Public Health and Preventive Medicine, Seoul National University Hospital, 101 Daehak-Ro Jongno-Gu, Seoul, Republic of Korea; 2 Department of Preventive Medicine, Seoul National University College of Medicine, 28 Yongon-Dong, Chongno-Gu, Seoul, 110-799 Republic of Korea; 3 Department of Food and Nutrition, Research Center for Human Ecology, College of Human Ecology, Kyung Hee University, 26 Kyungheedae-Ro Dongdaemun-Gu, Seoul, Republic of Korea; 4 Institute of Environmental Medicine, Seoul National University Medical Research Center, 103 Daehak-Ro Jongno-Gu, Seoul, Republic of Korea; 5 Environmental Health Center, Seoul National University College of Medicine, 103 Daehak-Ro Jongno-Gu, Seoul, Republic of Korea

**Keywords:** Cohort study, Dietary calcium, Serum calcium, Type 2 diabetes mellitus

## Abstract

**Background:**

Previous evidence regarding the associations between serum calcium concentrations, dietary calcium intake, and type 2 diabetes (T2D) is limited. We investigated the longitudinal associations of serum calcium levels and dietary calcium intake with T2D development.

**Methods:**

This study used data from the Ansung–Ansan cohort, a community-based, prospective cohort that was followed up for 10 years. Cox regression models adjusted for potential confounders were used to evaluate the associations of serum calcium levels (mean, 9.41 mg/dL) and dietary calcium intake (median, 389.59 mg/day) with T2D incidence. Association between dietary calcium intake and serum calcium levels was assessed using linear regression models.

**Results:**

Albumin-adjusted serum calcium levels were not associated with T2D risk (hazard ratio [HR] = 1.07, 95% confidence interval [CI] 0.96, 1.19, *p*-value = 0.2333). A one-unit increase in log-transformed, energy-adjusted dietary calcium intake was associated with a decreased risk of T2D (HR = 0.88, 95% CI 0.77, 1.00, *p*-value = 0.0460) and lower albumin-adjusted serum calcium levels (*β* = − 0.04, 95% CI − 0.07, − 0.02, *p*-value = 0.0014). The associations did not differ according to sex (all *p*-values for interaction > 0.10).

**Conclusions:**

Serum calcium levels were not associated with T2D risk, while higher dietary calcium intake was associated with a decreased risk of T2D development. These results have public health implications for predicting and preventing T2D development, as well as providing guidelines for diet and calcium supplementation.

## Background

Type 2 diabetes (T2D) is one of the most important public health issues worldwide, with the past few decades witnessing a rapid increase in the global number of affected patients [1]. Recently, limited number of studies have reported that higher serum calcium levels are associated with an increased risk of T2D development [2–5]. However, the association between serum calcium levels and T2D development was not observed in another epidemiological study [6]. In addition, the positive associations between serum calcium levels and T2D were unexpected, considering that higher calcium intake has been associated with lower T2D risk [7, 8].

Because the previous evidences on the associations between serum calcium concentrations, dietary calcium intake, and T2D development are inconsistent and limited, further investigation into these complex relationships are needed for predicting and preventing T2D development and providing scientific guidelines for diet and/or calcium supplementation in the general population.

Therefore, in the present study, we evaluated the longitudinal associations of serum calcium levels and dietary calcium intake with incidence of T2D in a community-based, prospective cohort, followed up for 10 years. We also assessed the association between dietary calcium intake and serum calcium levels at the baseline survey.

## Methods

### Study design and population

The present study was conducted using data from the Ansung–Ansan cohort, an ongoing community-based, prospective cohort. Detailed information on the Ansung–Ansan cohort are presented elsewhere [9]. In brief, 10,038 participants aged 40–69 years who resided in the Ansung or Ansan regions of the Republic of Korea were recruited between 2001 and 2003 using a two-stage cluster sampling method. Follow-up surveys were conducted biennially. During each survey, participants took part in interviews using structured questionnaires, health examinations, and laboratory tests.

Among the 10,038 participants in the Ansung–Ansan cohort, 676 were excluded from analysis because they reported at enrollment that they had been previously diagnosed with T2D. In addition, 560 participants were excluded from analysis because they had prevalent T2D during the baseline survey, as determined by predefined criteria, based on fasting glucose concentration (≥ 126 mg/dL), post-load glucose concentration (≥ 200 mg/dL), and antidiabetic medication use. Two participants who lacked information on serum calcium levels were also excluded, resulting in the inclusion of 8800 participants in the final analysis. In summary, the exclusion criteria of the present study was presence of T2D and absence of serum calcium levels, and therefore, individuals with hypertension, dyslipidemia, or chronic kidney disease were included in the analysis. The present study used a sequential modeling approach and conducted several analyses with different covariate sets. Therefore, the number of samples used for each analysis was different between models, and this information, along with the results, is presented.

The Ansung–Ansan study protocol was reviewed and approved by the Institutional Review Board of the Korea Centers for Disease Control and Prevention, and all study participants submitted written informed consent. The present study was approved by the Ethical Review Board of Seoul National University Hospital (C-1306-046-495).

### Serum and dietary calcium assessment

Serum calcium and albumin were enzymatically measured at the baseline survey using a 747 Chemistry Analyzer (Hitachi, Tokyo, Japan). Since total calcium levels may vary with serum albumin levels due to calcium-albumin binding, serum calcium concentrations were adjusted for albumin concentrations [2] using the following equation:

$$ {\text{Albumin-adjusted serum calcium }}\left( {{\text{mg}}/{\text{dL}}} \right) = {\text{serum calcium }}\left( {{\text{mg}}/{\text{dL}}} \right) + \left[ {0.8 \times \left( {4{-}{\text{albumin }}\left( {{\text{g}}/{\text{dL}}} \right)} \right)} \right]. $$

The adjusted level was then used as an explanatory variable in the analysis.

Dietary data were collected during the baseline survey by trained interviewers using a validated semi-quantitative food frequency questionnaire [10]. The study participants reported the average consumption frequency and portion size of 103 food items during the previous years, and daily nutrient and total energy intake were calculated using the dietary habit information, as well as the nutrient and energy content for each food item. To control for potential confounding by energy intake, daily calcium intake was adjusted by total energy intake using the residual method [11]. Since the distribution of energy-adjusted calcium intake was highly skewed, log-transformed values were used in the analysis. The same analyses were also conducted using log-transformed calcium intake not adjusted for total energy intake, to assess the robustness of the results.

### Definition of T2D

During each survey, a 75-g oral glucose tolerance test was performed for participants who were not known to have T2D and had not started diabetes treatment since the last survey. T2D was defined as any of the following conditions: fasting glucose concentration ≥ 126 mg/dL, post-load 2-h glucose concentration ≥ 200 mg/dL, or antidiabetic medication use. In the present analysis, data for T2D detection was available for every survey from the baseline survey conducted in 2001–2003, up to the fifth follow-up survey conducted in 2011–2012.

### Statistical analysis

The associations of albumin-adjusted serum calcium levels and energy-adjusted dietary calcium intake with T2D development were assessed using the Cox proportional hazards models with age as the time scale. The potential nonlinearity of the association was assessed through nonparametric analysis that applied spline smoothing methods [12].

We constructed analytical models by including covariates assessed at the baseline survey sequentially, to confirm robustness of the results. We selected the covariates based on previous literature and assumed biological pathways [2–5, 13]: (1) adjusted for age and sex; (2) further adjusted for residential area (Ansung or Ansan), monthly family income (< $869, $869–1738, $1738–3475, or ≥ $3475), tobacco smoking (non-smoker, ex-smoker, current smoker), alcohol intake (non-drinker, ex-drinker, or current drinker), physical activity (none, < 3, or ≥ 3 episodes/week, with each episode defined as exercising for more than 30 min), and body mass index (< 18.5, 18.5–25, 25–30, or ≥ 30; weight divided by height squared, kg/m^2^); (3) further adjusted for systolic blood pressure (mmHg), diastolic blood pressure (mmHg), and serum creatinine level (mg/dL). Finally, we constructed the analytical model mutually adjusted for serum calcium levels and dietary calcium intake, by including terms for serum calcium levels, dietary calcium intake, and above-mentioned covariates. To approximate the normal distribution, log-transformed values of dietary calcium intake and serum creatinine level were used in the analysis.

We assessed the cross-sectional association between dietary calcium intake and serum calcium levels at the baseline survey by linear regression models adjusted for the same covariate sets.

Potential heterogeneity of the associations of serum calcium levels and dietary calcium intake with T2D according to sex was explored because many of the previous studies only examined the association of calcium intake with T2D in women [7, 8, 14]. Interactions with sex were evaluated with the likelihood ratio test, by testing the product terms between sex and serum calcium levels or dietary calcium intake added to the main models.

We evaluated the robustness of the results by excluding those who had used diuretics or those who were using diuretics, because diuretics could also affect serum calcium levels.

All analyses were conducted using SAS version 9.4 (SAS Institute Inc., Cary, NC) and R version 3.2.5 (The Comprehensive R Archive Network, Vienna, Austria: http://cran.r-project.org).

## Results

Baseline participant characteristics and associated serum calcium levels are presented in Table 1. The mean age at enrollment was 51.8 years, and there was a slightly higher proportion of women (53%) than men (47%). Majority of the participants were non-smokers (59%), current drinkers (47%), and did not exercise regularly (75%). The mean body mass index was 24.5 kg/m^2^. The mean (standard deviation) albumin-adjusted serum calcium level was 9.41 (0.52) mg/dL (first quartile, 9.12 mg/dL; median, 9.48 mg/dL; third quartile, 9.76 mg/dL). The median (interquartile range) dietary calcium intake was 389.59 (283.71) mg/day (first quartile, 268.94 mg/day; third quartile, 552.65 mg/day).

**Table 1:** Baseline characteristics and albumin-adjusted serum calcium concentrations of the study participants (*n* = 8800)

Characteristics	Values^a^	Calcium^b^, mean (SD)	*p*-value^c^
Sex			< 0.0001
Men	4105 (47)	9.38 (0.56)	
Women	4695 (53)	9.44 (0.48)	
Area			< 0.0001
Ansung	4358 (50)	9.44 (0.49)	
Ansan	4442 (50)	9.38 (0.54)	
Monthly family income			< 0.0001
< $869	3000 (35)	9.48 (0.49)	
$869–$1738	2561 (30)	9.41 (0.50)	
$1738–$3475	2443 (28)	9.35 (0.54)	
≥ $3475	634 (7)	9.24 (0.56)	
Tobacco smoking			< 0.0001
Non-smoker	5160 (59)	9.42 (0.49)	
Ex-smoker	1305 (15)	9.34 (0.56)	
Current smoker	2216 (26)	9.40 (0.55)	
Alcohol intake			< 0.0001
Non-drinker	4036 (46)	9.43 (0.49)	
Ex-drinker	546 (6)	9.42 (0.53)	
Current drinker	4137 (47)	9.38 (0.54)	
Physical activity			0.0014
None	6629 (75)	9.42 (0.52)	
< 3 episodes/week	428 (5)	9.35 (0.53)	
≥ 3 episodes/week	1743 (20)	9.38 (0.51)	
Body mass index (kg/m^2^)			0.0005
< 18.5	167 (2)	9.42 (0.51)	
18.5–25	4997 (57)	9.39 (0.52)	
25–30	3225 (37)	9.43 (0.52)	
≥ 30	407 (5)	9.48 (0.49)	
Age (years)	51.8 (8.8)		< 0.0001
Systolic blood pressure (mmHg)	120.1 (18.8)		< 0.0001
Diastolic blood pressure (mmHg)	79.6 (12.1)		0.0073
Estimated glomerular filtration rate (mL/min/1.73 m^2^)	73.7 (13.9)		< 0.0001
HOMA-IR	1.3 (1.9)		0.0049
Insulinogenic index	0.4 (3.9)		0.0247
Fasting glucose (mg/dL)	83.0 (8.8)		< 0.0001
Post-load 2-h glucose (mg/dL)	115.3 (30.9)		0.4335
HbA1c (%)	5.6 (0.4)		< 0.0001

*SD* standard deviation, *HOMA-IR* homeostatic model assessment-insulin resistance index, *HbA1c* glycated hemoglobin

^a^Values are presented as *n* (%) for categorical variables, mean (standard deviation) for age, systolic blood pressure, diastolic blood pressure, estimated glomerular filtration rate, fasting glucose level, post-load 2-h glucose level, and Hb1Ac or geometric mean (geometric standard deviation) for the other continuous variables

^b^Albumin-adjusted serum calcium concentrations (mg/dL)

^c^*p*-values are estimated by one-way analysis of variance for categorical variables and bivariate linear regression analysis for continuous variables

**Fig. 1:**
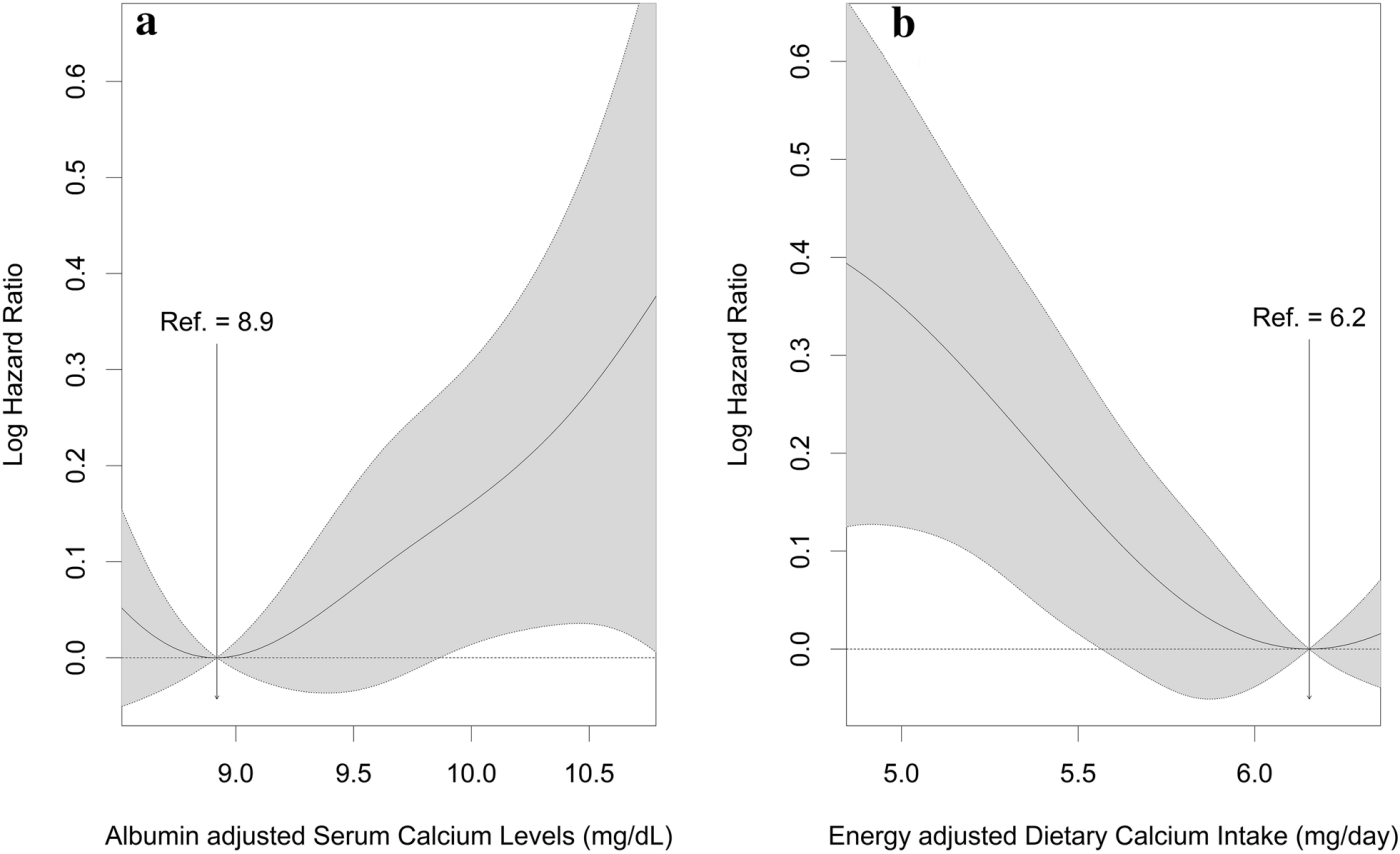
Penalized regression spline model for the associations of albumin-adjusted serum calcium levels and energy-adjusted dietary calcium intake with incidence of type 2 diabetes. **a** Association between albumin-adjusted serum calcium intake and risk of type 2 diabetes. **b** Association between energy-adjusted dietary calcium intake and risk of type 2 diabetes. Solid line, spline curve; shaded area, 95% confidence interval. The models were adjusted for age, sex, residential area, monthly family income, tobacco smoking, alcohol intake, physical activity, body mass index, systolic blood pressure, diastolic blood pressure, and serum creatinine level

The associations of serum calcium levels and dietary calcium intake with T2D were found to be robust in penalized regression spline models (Fig. 1). In the fully-adjusted Cox proportional hazard models, we did not find the evidence for the association between serum calcium levels and risk of T2D (Table 2). However, a one-unit increase in log-transformed energy-adjusted dietary calcium intake was associated with a lower risk of T2D (Table 3). The associations between dietary calcium intake and T2D did not change appreciably between models not adjusted for serum calcium levels (hazard ratio [HR] = 0.87, 95% confidence interval [CI] 0.77, 0.99) and models adjusted for serum calcium levels (HR = 0.88, 95% CI 0.77, 1.00). These results were robust in analyses using dietary calcium not adjusted for total energy intake (data not shown).

**Table 2:** Association between albumin-adjusted serum calcium levels and type 2 diabetes in the Ansung–Ansan cohort of the Korean Genome and Epidemiology Study (KoGES)

	*n* ^a^	Hazard ratio	95% confidence interval	*p*-value
Model 1^b^	8800	1.10	0.99, 1.22	0.0919
Model 2^c^	8534	1.10	0.98, 1.22	0.0947
Model 3^d^	8525	1.07	0.96, 1.19	0.2125
Model 4^e^	8433	1.07	0.96, 1.19	0.2333

^a^Number of study participants included in each analysis

^b^Adjusted for age and sex

^c^Adjusted as in Model 1, and further adjusted for residential area, monthly family income, tobacco smoking, alcohol intake, physical activity, and body mass index

^d^Adjusted as in Model 2, and further adjusted for systolic blood pressure, diastolic blood pressure, and serum creatinine level

^e^Adjusted as in Model 3, and further adjusted for dietary calcium intake

**Table 3:** Association between dietary calcium intake and type 2 diabetes in the Ansung–Ansan cohort of the Korean Genome and Epidemiology Study (KoGES)

	*n* ^a^	Hazard ratio	95% confidence interval	*p*-value
Model 1^b^	8685	0.90	0.80, 1.02	0.0900
Model 2^c^	8444	0.87	0.77, 0.99	0.0359
Model 3^d^	8433	0.87	0.77, 0.99	0.0390
Model 4^e^	8433	0.88	0.77, 1.00	0.0460

^a^Number of study participants included in each analysis

^b^Adjusted for age and sex

^c^Adjusted as in Model 1, and further adjusted for residential area, monthly family income, tobacco smoking, alcohol intake, physical activity, and body mass index

^d^Adjusted as in Model 2, and further adjusted for systolic blood pressure, diastolic blood pressure, and serum creatinine level

^e^Adjusted as in Model 3, and further adjusted for serum calcium level

When we assessed the association between dietary calcium intake and serum calcium levels at the baseline survey by linear regression model, dietary calcium intake was inversely associated with serum calcium levels after adjusting for potential confounders (*β* = − 0.04, 95% CI − 0.07, − 0.02).

The associations of serum calcium levels and dietary calcium intake with T2D did not differ according to sex (*p* > 0.10 for all interactions).

After excluding those who had used diuretics (14 participants, 0.16%) or those who were using diuretics at the baseline survey (8 participants, 0.09%), the results were almost similar.

## Discussion

In the present study with a community-based cohort followed-up for 10 years, the association between serum calcium levels and a risk of incident T2D was not evident, while higher dietary calcium intake was associated with a decreased risk of incident T2D.

Previous studies on the association between serum calcium levels and T2D have demonstrated inconsistent results. In a population-based prospective cohort study conducted in Norway, higher serum calcium concentrations were associated with an increased risk of incident T2D [3]. In a multicenter epidemiological study in the United States, in which participants were surveyed twice at an average of 5.2-year intervals, serum calcium levels from the first survey predicted an increased detection of T2D in the second survey [4]. In another prospective cohort study conducted among individuals with high cardiovascular risk in Spain, higher serum calcium levels were associated with T2D risk [2]. In a retrospective cohort study in China, elevated serum calcium levels were also associated with T2D risk [5]. However, in a prospective cohort study in Finland with a median follow-up of 23.1 years, levels of ionized calcium, a direct measurement of active serum calcium, were not associated with a T2D risk. The reason for the inconsistency in the results is not clear. However, differences in population characteristics such as race, genetic background, and vitamin D, parathyroid, and phosphorus levels might be responsible for the inconsistency. In addition, difference in measurement errors in exposure and outcome and different covariates adjusted in analytical models might also contribute to the inconsistency. Because the number of studies exploring this association is limited, further studies, especially those using direct measure of active calcium, are warranted.

Four large cohort studies have investigated the association between calcium intake and T2D in the United States (*n* = 83,779 [7], (*n* = 41,186) [14]), China (*n* = 64,191) [8], and Japan (*n* = 59,796) [15]. These studies reported inverse associations between dietary or total calcium intake and T2D risk among women in the United States, women in China, or individuals with higher vitamin D intake in Japan. Another cohort study conducted in rural areas in Korea (*n* = 8313) also reported an inverse association between total and vegetable calcium intake, and T2D risk among women [16]. However, a relatively small study (*n* = 5200) in Australia found no association between dietary calcium and T2D [17]. Additionally, studies provided mixed results when investigating the association between dairy products (a key dietary calcium source) and T2D. One confounder could be the high fat content in some dairy products, which may mitigate the protective effects of calcium [8, 18]. Calcium intake may also depend on non-dairy foods, including tofu, fish, rice, vegetables, and legumes, so the main source of dietary calcium may differ across populations and cultures [8]. Thus, geographically diverse studies may help to evaluate the association between calcium intake and T2D by reducing the effects of this potential confounder. However, possibility that the observed inverse association between dietary calcium intake and T2D risk might be attributable to dietary source of calcium still remains.

In the present study, serum calcium levels were not associated with T2D risk, while dietary calcium intake was inversely associated with T2D risk. This might be because serum calcium levels could reflect not only exogenous calcium intake but also the ability to maintain homeostasis.

Dietary calcium intake was inversely associated with serum calcium levels. Although vitamin D insufficiency is highly prevalent in the Republic of Korea [19] and lower calcium intake was reported to be associated with an increased parathyroid hormone levels in individuals with low serum vitamin D levels [20], to our knowledge, there has been few study directly investigating these inverse association and underlying mechanisms. Further studies using the information on vitamin D or parathyroid hormone are warranted.

Calcium reportedly functions in intracellular processes mediated by insulin in skeletal muscle and adipose tissue, and potentially affects insulin sensitivity in these insulin-responsive tissues [21, 22]. Calcium is also essential for insulin secretion from pancreatic β-cells in response to elevated blood glucose levels, acting via voltage-gated calcium channels or large transient receptor potential channels [23]. Increased calcium intake may also decrease the risk of osteoporosis and reduce the release of environmental pollutants such as lead, which are sequestered in bone and can increase insulin resistance and T2D risk [24]. In addition, an inverse association between dietary calcium intake and a risk of T2D may be indirectly attributable to changes in gastrointestinal hormones or intestinal microbiota and integrity [25].

There are several limitations of the present study. First, although ionized calcium is physiologically active and a measurable biomarker of calcium homeostasis, the pertinent data were not available. Instead, the present study used albumin-adjusted serum calcium levels, which is highly correlated with ionized calcium with large sample sizes [26], to consider variation in total calcium levels according to serum albumin [2, 4, 5]. Second, although vitamin D or parathyroid hormone may affect the association between serum calcium levels and T2D, we had no information available regarding these biomarkers. This limited our ability to comprehensively evaluate the associations between calcium, vitamin D, parathyroid hormone, and T2D. Third, calcium supplement information was lacking, which could result in misclassification of total exogenous calcium intake.

However, the present study also has considerable strengths. The study used data from a well-designed, community-based prospective cohort that was followed up for 10 years. The data included various clinical, dietary, and epidemiological traits; they provided a unique opportunity to investigate the complex association between serum calcium levels, dietary calcium intake, and incident T2D.

## Conclusions

Serum calcium levels were not associated with a T2D risk, but higher dietary calcium intake was associated with a decreased T2D risk. The present results have potential public health implications for predicting and preventing T2D development, as well as providing guidelines for diet and calcium supplementation. Because previous evidence regarding the association between serum calcium levels, dietary calcium intake, and T2D are insufficient, further studies conducted among different populations are warranted to confirm the present results.

## Abbreviations

CI: confidence interval
HR: hazard ratio
T2D: type 2 diabetes
